# Crosstalk between gut microbiota and host immune system and its response to traumatic injury

**DOI:** 10.3389/fimmu.2024.1413485

**Published:** 2024-07-31

**Authors:** Hanif Ullah, Safia Arbab, Yali Tian, Yuwen Chen, Chang-qing Liu, Qijie Li, Ka Li

**Affiliations:** ^1^ Medicine and Engineering Interdisciplinary Research Laboratory of Nursing & Materials/Nursing Key Laboratory of Sichuan Province, West China Hospital, Sichuan University/West China School of Nursing, Sichuan University, Chengdu, Sichuan, China; ^2^ Lanzhou Institute of Husbandry and Pharmaceutical Sciences, Chinese Academy of Agricultural Sciences, Lanzhou, China

**Keywords:** gut microbiota, immune system, traumatic injury, probiotic, dysbiosis

## Abstract

Millions of microorganisms make up the complex microbial ecosystem found in the human gut. The immune system’s interaction with the gut microbiota is essential for preventing inflammation and maintaining intestinal homeostasis. Numerous metabolic products that can cross-talk between immune cells and the gut epithelium are metabolized by the gut microbiota. Traumatic injury elicits a great and multifaceted immune response in the minutes after the initial offense, containing simultaneous pro- and anti-inflammatory responses. The development of innovative therapies that improve patient outcomes depends on the gut microbiota and immunological responses to trauma. The altered makeup of gut microbes, or gut dysbiosis, can also dysregulate immunological responses, resulting in inflammation. Major human diseases may become more common as a result of chronic dysbiosis and the translocation of bacteria and the products of their metabolism beyond the mucosal barrier. In this review, we briefly summarize the interactions between the gut microbiota and the immune system and human disease and their therapeutic probiotic formulations. We also discuss the immune response to traumatic injury.

## 1 Introduction

The interactions between the host immune system and the microbiota that live in the human gastrointestinal (GI) tract, known as the gut microbiota, constitute a dynamic area of research. Metabolites from the gut microbiota interact both directly and indirectly with host immune cells, playing important roles in inflammatory signaling (1). The absorption and digestion of nutrients are facilitated by gut microorganisms. By secreting digestive enzymes and boosting enzymatic activity, gut microorganisms contribute significantly to the host’s nutritional digestibility and aid in nutrient harvesting (2). Inflammation and host immunity are closely regulated by the gut microbiome. Dietary probiotics increase immunity and reduce host animal inflammatory reactions (3). The gastrointestinal tract is among the body’s important immunological organs (4). The intestinal barrier, which protects the host from infections, is a multilayer system made up of immunological, chemical, mechanical, and microbiological barriers (5). Certain bacteria metabolize complex carbohydrates to produce short-chain fatty acids (SCFAs), which influence host immune cells and provide colonocytes with carbon (6, 7). Traumatic injury initiates a complex and dynamic immune response within minutes of initial injury; these reactions are a direct result of tissue injury and hemorrhage instead of an infection (8). This “sterile inflammation” is usually caused by immune cell release of damage-associated molecular patterns (DAMPs) from necrotic and injured cells (9), cytokine release, and complement activation (10). Traumatic injury causes approximately 8% of all deaths worldwide each year, making it one of the most common causes of death worldwide (11). Trauma is caused by earthquakes, typhoons, fires, traffic accidents, falls from high altitudes, etc. (12). Furthermore, the cumulative incidence of severe trauma has been rising globally over the past decade (13). Immune system control, food metabolism, human growth and development, and other critical physiological processes are influenced by intestinal microbes (14). In both health and disease control, the relationship between the host and microbes is important. The diversity of the gut microbiota is strongly influenced by several host characteristics, including age, environment, nutrition, and lifestyle. Nonetheless, food is believed to be one of the main variables (modifiers) involved in adjusting the gut microbiota (15). The human microbiome exhibits promising potential for modifying malnutrition, enhancing nutrient uptake, and using energy from diverse food sources. Additionally, microbes are essential for the metabolism of xenobiotics. Different gut microorganisms change the chemical structures of medications, pollutants, and numerous insecticides during xenobiotic metabolism (16). An essential part of the immunological barrier is formed by diffuse immune cells and gut-associated lymphoid tissue (GALT). Because gut-associated lymphoid tissue can detect and scavenge harmful microorganisms, it can prevent abnormal immune responses and preserve the equilibrium of host immunity. The nuclear factor kappa-B (NF-kB) signaling pathway and Toll-like receptors (TLRs) are involved in the development of immunological tolerance (17, 18). Numerous studies have detailed the tremendous impact that commensal bacterial metabolites have on immune cells, including conventional T lymphocytes (adaptive response) and dendritic cells (innate immunity). However, the local presence of T cells also helps the immune system mount quick effector responses (19). The gut microbiota and host immunity have intricated, dynamic, and context-dependent relationships. Here, we summarize and highlight significant recent findings as well as fundamental ideas that connect the microbiome to immune system development and function. In addition, we discuss the challenges and potential benefits of using microbiome-targeted approaches to investigate the etiology of disease and the human immune system’s response to traumatic injury.

## 2 The gut microbiota and immune dysregulation

The gastrointestinal tract (GIT) harbors a substantial collection of microorganisms known as the gut microbiota. The human gut consists of a population of approximately 1000 species and 7000 different types of bacteria, mostly gram-negative Bacteroidetes (which include Bacteroides and Prevotella) and gram-positive or gram-negative Firmicutes (including the species Lactobacillus, Eubacterium, and Clostridium). The gut microbiota comprises genes that are 100–150 times more abundant than those found in the human genome (20–22). The majority of the gut microbial community is composed of the following five phyla: Verrucomicrobia, Actinobacteria, Proteobacteria, Firmicutes, and Bacteroidetes (22, 23). The bacterial population in the small intestine is quite low, particularly in the duodenum and jejunum. This is because of the relatively fast movement of the bacteria and the low pH caused by several digesting enzymes (24). Aerobes, facultative anaerobes, and anaerobes are among the organisms found in the gut. A total of 99% of the gut microbiota comprises anaerobes (25). The intestinal microbiota can be divided into three groups based on how they affect the gut (26), (1) physiological bacteria, which are the predominant anaerobic bacterial communities found in the gastrointestinal system and primarily consist of Bacteroides, Lactobacillus, and Bifidobacterium; (2) opportunistic pathogens, which include Klebsiella pneumonia and *Escherichia coli* and are primarily facultative aerobes; and (3) pathogenic bacteria, such as *Clostridium perfringens* (27). Within the human host, the gut microbiota functions as a “superorganism,” assisting in food absorption, generating metabolites that nourish the host, guarding against infection, preserving the structure and function of intestinal epithelial cells, and controlling host immunity (28, 29). In a state of balance known as “eubiosis,” the gut microbiota exists in healthy individuals. However, when a disease occurs, the gut microbiota enters an unbalanced state of dysbiosis when a decrease in helpful commensals, an increase in opportunistic pathogens, or both occur (30). During pregnancy, the embryo’s stomach inside the uterus is sterile (31). However, as soon as the baby comes into contact with the community, microbes begin to colonize the gastrointestinal system, creating a stable gut microbial population (32). Bifidobacterium species are among the first microorganisms to populate the gut. The newborn’s delivery, postnatal nutrition, gestational age, degree of hygiene, and medicine all have an impact on the dynamics of gut microbiome colonization (33). The gut microbiota composition is influenced by genetic factors, the environment, age, and food (34).

The gastrointestinal (GI) tract, also known as the digestive system, is a complex system responsible for breaking down food, absorbing nutrients, and eliminating waste products. It includes the mouth, esophagus, stomach, small intestine, large intestine (colon), and anus. The GI tract plays a vital role in maintaining overall health, and any disorders or diseases in this system can significantly impact quality of life. Some common conditions affecting the GI tract include irritable bowel syndrome (IBS), gastroesophageal Reflux Disease (GERD), Inflammatory Bowel Disease (IBD), Peptic Ulcer Disease, Gastroenteritis (inflammation of the stomach and intestines), and Cancer.

The intestine consists of multiple layers including the mucosa, submucosa, muscularis propria, and serosa. The mucosa is of particular interest in terms of the microbiome and includes the epithelium, lamina propria, and muscular mucosa (35). The inner most epithelial layer is held together by complex junctions. From the most superficial, these include tight junctions, adherens junctions, and desmosomes (36). Together, these prevent intestinal contents from translocating outside the gastrointestinal tract but can be affected by various disease states. The mucosa and gut epithelial cells act as physical barriers to prevent endotoxemia and infections. Short-chain fatty acids (SCFAs) and secondary bile acids (SBAs), two metabolites of the gut microbiota, control gut permeability through immunomodulation. Th1 differentiation and effector activity are enhanced in naïve T cells by the gut microbiota-derived metabolite inosine, which is generated by *Bifidobacterium* and *A. muciniphila* (37). Intestinal permeability is thought to be increased by gut microbiota dysbiosis due to a “leaky gut,” which permits opportunistic pathogens and their microbial products/toxins to enter the bloodstream and eventually mount an inflammatory response. Therefore, gut microbiota-mediated immune responses are critical for preventing intestinal permeability (38, 39). Numerous recognized metabolites, including those that are sulfur- and phenol-containing and that can damage intestinal epithelia, interfere with intercellular tight junctions (TJ), and promote bacterial translocation, provide support for this idea (40). These outcomes result in inflammatory diseases, immune cell dysfunction, and an inability to eradicate invasive pathogens (41). The intestinal microbiota has a role in the maturation and development of the early immune system. Dysregulation of the microbiota-gut-brain axis can result in metabolic, psychological, and neurological diseases (42).

Tight junction (TJ) proteins play a crucial role in maintaining intestinal permeability, which is essential for regulating the interactions between the microbial community in the gut and the immune system. TJ proteins, such as occludin, claudins, and junctional adhesion molecules (JAMs), form a barrier between intestinal epithelial cells, controlling the passage of molecules and ions through the paracellular pathway. When TJ proteins are disrupted or altered, intestinal permeability increases, allowing toxins, undigested food particles, and microorganisms to cross the epithelial barrier and interact with the immune system. This increased permeability can lead to, activation of immune cells, such as macrophages and dendritic cells, which can trigger pro-inflammatory responses, Increased antigen presentation, leading to an enhanced immune response and potentially contributing to autoimmune diseases. Changes in the gut microbiota composition, as altered permeability can influence the growth and survival of different microbial populations, and Production of pro-inflammatory cytokines, which can further exacerbate intestinal permeability and contribute to a vicious cycle of inflammation. Conversely, a healthy intestinal epithelial barrier, maintained by intact TJ proteins, prevent excessive microbial translocation and reduces immune system activation. Promote a balanced gut microbiota, supporting immune homeostasis and enhancing the production of anti-inflammatory cytokines, contributing to a tolerant immune environment.

## 3 Interaction between the gut microbiota and host immunity

The host’s innate and adaptive immune systems are trained and developed in particular by its microbiome, and the immune system coordinates the preservation of important aspects of host-microbe symbiosis (43). The gut microbiota, mesenteric lymph nodes, specialized epithelial cells, innate and adaptive immune cells, and related metabolites make up the majority of the intestinal immune system (44). The metabolites produced by gut microbes are important for inflammatory signals because they interact with host immune cells both directly and indirectly (1). Through intestinal epithelial cells, the gut microbiota and metabolites can control the growth and activity of the immune system (45). The immune system consists of two branches, innate and adaptive, which interact to defend the body against both internal and external threats. The “first line of defense” is the innate immune system, which responds quickly and broadly to an immunological stimulus. Granulocytes, natural killer cells, dendritic cells, and macrophages are components of innate immunity; they engulf pathogens and release cytokines and chemokines. Cytokines generate lymphocytes, such as B cells, which generate antibodies specific to the particular pathogenic insult, and T cells, which are primarily divided into helper T cells, cytotoxic T cells, and regulatory T cells (Treg cells). These cells are the foundation of adaptive immunity in addition to drawing in more innate immune cells (46).

### 3.1 The gut microbiota and innate immune system

#### 3.1.1 Gut-associated lymphoid tissues and the mucosal defense system

GALTs line the direct path between the host and the environment and are a component of mucosa-associated lymphoid tissues (MALTs). Innate immune cells in GALTs serve as the first line of defense for the gut mucosa. The primary responsibilities of immune cells include nonspecifically identifying pathogens, triggering the innate immune response, and presenting antigens to trigger the adaptive immune system downstream. GALTs play a critical role in maintaining immunological tolerance to commensal bacteria. The balance between the human immune system and the gut microbiota depends on the dual role of GALTs.

**Figure 1:**
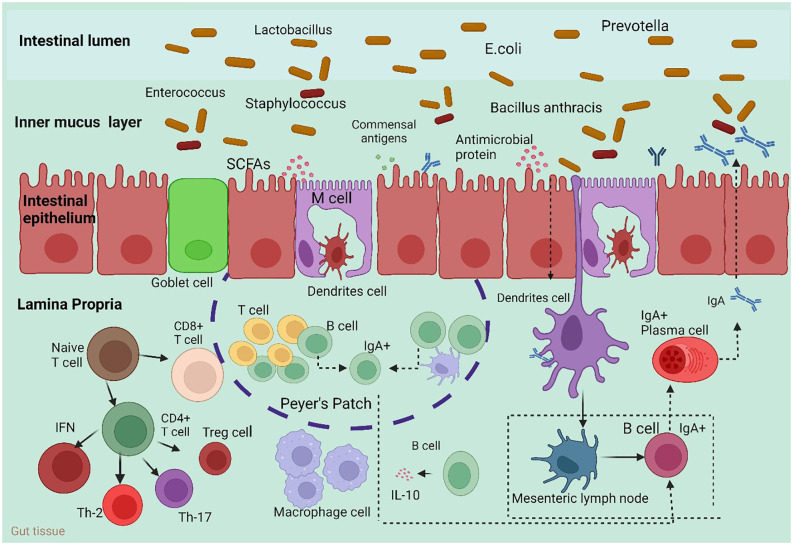
Host immune responses to the intestinal microbiota. To maintain intestinal homeostasis, a number of immune mechanisms interact with gut bacteria in harmony. Through Toll-like receptors (TLRs), goblet cells secrete mucin glycoproteins, plasma cells release IgA, and epithelial cells secrete antimicrobial proteins. After migrating to Peyer’s patches and mesenteric lymph nodes, where B cells develop into IgA-secreting plasma cells, dendritic cells (DCs) absorb microorganisms. Furthermore, intestinal DCs isolated from Bacteroides fragilis polysaccharide A (PSA) induce regulatory T (Treg) cells, which are responsible for producing IL-10. Furthermore, the microbiota composition can be altered by antimicrobial proteins secreted by host cells. M cell; interleukin 10 (IL-10). Macrophages carry out their regular duties after migrating to the digestive tract lamina propria.

The mesenteric lymph nodes (mLNs), appendix, isolated lymphoid follicles (ILFs), Peyer’s patches, and crypt patches are the principal histological components of GALTs (**Figure 1**) (47). M cells, one of the constituent cells of GALTs, are capable of transferring antigens but cannot process or display them (48); conventional lymphocytes, such as helper T cells (Th cells) (49); and additional atypical lymphocytes, such as innate lymphoid cells (ILCs) (50). The innate immune cells include membranous cells, neutrophils, eosinophils, basophils, natural killer cells, and macrophages. In particular, neutrophils and macrophages may consume pathogens because of their phagocytic activity. Innate immune molecules contain complement, lysozyme, and interferon (IFN). Nucleotide binding, oligomerization domain-like receptors, Toll-like receptors (TLRs), and other pattern recognition receptors are necessary for the activation of nonspecific host immunity (51). Several studies have revealed that the gut microbiota is necessary for the structural assembly of GALTs. The systemic immune response and the local immunological response to the gut microbiota are largely dependent on GALTs. Through Pattern Recognition Receptors (PRR-PAMP recognition and epigenetic modulators such as SCFAs, the gut microbiota changes the structural development of GALTs and primes their immunological response to activate host defense functions and maintain tolerance against commensal bacteria. In the pathophysiology of autoimmune illnesses, GALTs—particularly mesenteric lymph nodes—are the first to trigger gut-driven immune responses, which may have changed the immune response at the systemic level (52).

To protect the host body from infections, redundant systems make up the defense mechanisms. The two primary PRRs that are involved in the initial recognition of the gut microbiota are Toll-like receptors (TLRs) and nucleotide-binding oligomerization domain (NOD) molecules (53). The primary and most researched class of pattern recognition receptors (PRRs) is the Toll-like receptor (TLR) family. In the intestine, PRRs are extensively expressed on and in IECs, as well as in macrophages and dendritic cells (DCs). Pathogen- associated molecular patterns (PAMPs) on both pathogens and commensals are recognized by PRRs (54). An adequate immune response to a microorganism is triggered once it has been identified, internalized, or entered the epithelial layer (53). A plethora of intracellular signaling pathways, such as those involving kinases, transcription factors, and adaptor molecules, are activated by PRRs upon recognition of PAMPs. This signaling induces infection in the host and initiates proinflammatory and antimicrobial responses. Signal transduction pathways caused by PRR ultimately lead to the activation of gene expression and the manufacture of various molecules such as cytokines, chemokines, cell adhesion molecules, and immunoreceptors. The protective effects of proinflammatory cytokines, such as IL-8, IL-12, and IL-23, are mediated by the downregulation of these cytokines, while T regs produce anti-inflammatory cytokines, such as IL-10 (55, 56). DCs expose naive T cells to antigens, and the release of anti-inflammatory cytokines initiates both local and systemic tolerance (57). IECs, immune cells, and metabolites of the intestinal flora can all mediate crosstalk between the innate immune system and the intestinal flora. By binding to g protein-coupled receptors (GPCRs), SCFAs can control the release of inflammatory and anti-inflammatory compounds, such as TNF-α, from immune cells and IECs. Anti-inflammatory molecules include β-defensins. Moreover, SCFAs influence IECs via a variety of signaling pathways to promote the formation of mucus layers. By binding to GPR131 and the farnesoid X receptor (FXR), secondary bile acids (SBAs) control the expression of immunological chemicals, including antimicrobial peptides, by macrophages and IECs. By attaching to the pregnane X receptor (PXR) and the aryl hydrocarbon receptor (AhR), tryptophan metabolites regulate the release of immunological chemicals such as IL-22 by IECs and ILC3s (58). Through GPR109a, SCFAs also promoted the differentiation of T cells into Treg cells and inhibited the growth of Th17 cells by causing the production of IL-10 and aldehyde dehydrogenase 1a1 (Aldh1a1) in intestinal macrophages and dendritic cells (DCs) (59). Furthermore, valeric acid decreases the expression of IL-17a, which supports intestinal homeostasis, and increases IL-10 production in Th17 cells by enhancing glycolysis through HDAC inhibition (60). Butyrate increases Th1 cell expression of B lymphocyte-induced maturation protein 1 (Blimp-1), which stimulates the production of IL-10 and reduces the propensity of Th1 cells for inflammation. These effects are mediated by GPR43 and activate the STAT3 and mammalian target of rapamycin (mTOR) pathways (61).

#### 3.1.2 Innate lymphoid cells

The innate compartment of GALTs is composed primarily of innate lymphoid cells (ILCs). Recombinant activating gene (RAG)-mediated antigen receptor gene rearrangements are not necessary for the generation of ILCs, which is the primary characteristic that sets ILCs apart from T or B cells (62). T cells mediate cell-mediated immunity, while B cells mediate immunity. B cells can become memory B cells and plasma cells after becoming activated by an antigen; plasma cells can then release antibodies. Like immunoglobulins, antibodies include IgA, IgD, IgE, IgG, and IgM. Helper T cells (Th cells), memory T cells, cytotoxic T cells (Tc cells), and regulatory T cells (Treg cells or Tregs) are the different types of T cells. Target cells are attacked by cytotoxic T lymphocytes, which then cause the target cells to undergo apoptosis. Through the secretion of many cytokines, helper T cells can assist Tc and B cells in performing immune responses. Treg cells modulate the immunological response. Due to its influence on T-cell activation and the regulation of IgA secretion, the gut microbiome plays a role in the control of particular immune systems (63). The development of B cells, which are responsible for producing antibodies, is facilitated by the gut microbiota. Short-chain fatty acids, which are products of the gut microbiota, promote the development of B cells into cells that produce antibodies (64). Flagellin from symbiotic strains stimulates the production of retinoic acid, which can cause B-cell differentiation (65). There are three main subpopulations of ILCs. Like Th1 cells, Group 1 ILCs secrete the signature cytokine interferon (IFN)-γ in a manner dependent on T-bet or TBX21 (66). Group 2 ILCs are distinguished from Th2 cells by their reliance on GATA3 and their capacity to release IL-5 and IL-13 (67–69). Group 3 ILCs release the indicator cytokines IL-17 and IL-22 and, like Th17 and Th22 cells, need on the transcription factor RORγt (69). Group 3 ILCs consist of the CD4+ CD3– CCR6+ subset, namely, LTi cells, and the ILC3 subpopulation that does not express the tissue homing factor CCR6 (70).

#### 3.1.3 Natural killer cells

Conventional natural killer (cNK) cells are known as the only cytotoxic population of ILCs. The ability of these cells to discriminate between “nonself” and “self” through the signaling pathway system, which comprises activating and inhibitory receptors, makes them special (71). The cNK cell pool comprises a tissue-resident compartment and a circulating compartment. A considerable fraction of circulating cNK cells is found in the blood and primary lymphoid organs, such as the spleen and bone marrow. The majority of the biological characteristics of the tissue-resident cNK cell compartments in the gut intraepithelial layer and lamina propria layer are shared with other cNK cell compartments (72). In contrast to circulating cNK cells, these cNK cells have greater cytotoxicity and cytokine generation capacity. The pathophysiology of autoimmune disorders is unclear in relation to the importance of the variable cNK cell phenotype between the afflicted tissue and periphery. However, comparing these cNK cells to circulating cNK cells revealed greater cytotoxicity and cytokine generation capacity. However, the importance of the variable cNK cell phenotype between the afflicted tissue and periphery is still unknown (73). Future research is needed to determine the precise role that cNK cells play in the pathophysiology of autoimmune disorders, particularly to about the activating and inhibitory receptors that are influenced by the gut microbiota.

#### 3.1.4 Phagocytes: macrophages and dendritic cells

The immune cell population that is closely associated with the gut microbiota consists of phagocytes, which include macrophages and dendritic cells, as well as other nonimmune cells, such as IECs, that can present antigens and carry out phagocytosis. The immune system’s ability to recognize pathogenic microbes and tolerate symbiotic bacteria is greatly aided by gut phagocytes, which are essential for maintaining gut homeostasis.

The resident macrophages in the gastrointestinal tract are notable for their tissue residency. The lamina propria, intraepithelial layer, epithelial layer, and GALT feature Peyer’s patches, isolated lymphoid follicles, and mLNs are the primary locations of gut macrophages. A significant fraction of gut macrophages (Tim-4- macrophages) are produced directly from circulating monocytes, in contrast to tissue-resident macrophages in other organs, such as the skin or liver (74). Moreover, the gut microbiota may influence the primitive hematopoiesis of myeloid cells in the yolk sac early on and in the bone marrow later on, which may facilitate the development of peripheral myelocytes, including macrophages. Due to a compromised host defensive immunological response mediated by myeloid cells, the absence of gut commensal flora greatly increases vulnerability to bacterial infection (75).

Dendritic cells share some of the features of macrophages, such as their distribution in the gut and phagocytosis. However, dendritic cells are special because of their potent capacity to present antigens to the adaptive immune system and digest them. Two subsets of gut-dendritic cells can be distinguished by differences in the expression of CD11b, the chemokine receptor CX3 CR1, and CD103 (αE integrin) (76). The presence of gut commensal flora allows CD103+ dendritic cells to migrate from the intestine to mLNs, where they can then stimulate T-cell migration to the gut lumen to initiate an immune response through chemotaxis, which is dependent on CCR7 (77). Another unique type of dendritic cell, CD103–CD11b+ CX3 CR1+, resembles macrophages but is less effective at activating T cells and migrating. Nonetheless, this particular subset of dendritic cells is thought to possess the ability to generate transepithelial dendrites, engulf invasive enteric pathogens, and subsequently present and process antigens (78). However, when there is a dysbiosis of the gut microbiota, as in the Salmonella infection model, CD103+ dendritic cells congregate in the enteric epithelium layer and form trans-epithelial dendrites to phagocytose pathogenic bacteria (79). However, research using the Myd88-knockout mouse model, the antibiotic-induced colitis model, and the dextran sulfate sodium (DSS)-induced colitis model all led to the same conclusion: CX3 CR1+ dendritic cells show a great ability to migrate to mLNs and present antigens (80). Dysbiosis, on the other hand, may disrupt the balance of the immune system, leading to the presentation of self- or commensal flora-associated antigens in an improper way that exacerbates disease.

### 3.2 The gut microbiota and adaptive immunity

Adaptive T cells are the primary contributors to cellular immunity and protect the host’s homeostasis from immune-mediated inflammatory diseases. The gut microbiota can promote T-cell development, enabling T cells to quickly react to signals from the intestinal lumen environment and mount adaptive immune responses (81).

#### 3.2.1 Gut microbiota CD8+ T and CD4+ T cells

T cell growth, function, and differentiation are impacted by the commensal microbiota to maintain host immunological homeostasis. T cells, which are divided into CD4+ and CD8+ T cells, are primarily responsible for regulating adaptive immune responses. Cytotoxic CD8+ T-cell activity and Th cell polarization are induced by a particular commensal microbiome (82). Nonetheless, upon monoclonal colonization, the intestine adaptive immune phenotype is severely disrupted by a particular strain of *Fusobacterium varium*. Compared to other bacteria, *F. varium* induced a higher frequency of colonic double-negative cells (CD4−CD8−TCRb+) and dramatically decreased the populations of CD4+ and CD8+ T cells. Furthermore, *F. varium* significantly inhibited a broad set of genes involved in the metabolism of bile acids, which has been demonstrated to be intimately linked to immunological function (83). A recent study demonstrated that the microbiome functions as an antigen for T cells and that some microorganisms, such segmented filamentous bacteria (SFB), aid in the formation of thymic T cells that are specific to the microbiota. These cells are classified as CD4+ T cells and CD8+ T cells, and their primary function is to regulate adaptive immune responses (84). Immune protection against intracellular pathogens, such as viruses and bacteria, as well as tumor surveillance, depend mainly on CD8+ T cells (85). Recent research has shown that CD8+ T-cell activity is mediated by microbial metabolites. Two important SCFAs, butyrate and propionate, limit the activation of CD8+ T lymphocytes via regulating the synthesis of IL-12 through antigen-presenting cells (APCs) (86). According to Luu et al., pentanoate generated by *Megasphaera (M.) massiliensis* stimulates effector CD8+ T-cell activity. Adoptive T-cell treatment was found to be more effective when *M. massiliensis* was present, as evidenced by higher expression of TNFα and IFNγ (87). The innate and adaptive immune systems’ wide range of immunological responses are controlled by natural killer T (NKT) cells. By releasing cytokines, they have the ability to destroy target cells and alter the immune response (88). Sphingomonas species are representative NKT cell stimulators and are gram-negative bacteria that are primarily found in the natural environment. NKT cell activation and IFNγ release are stimulated by glycosphingolipids and glycosylceramides from Sphingomonas because they function as microbial antigens (89). By inhibiting NKT cell growth in neonatal mice, sphingolipids made by B. fragilis regulate homeostasis. This suggests that the NKT cell-microbiota network is essential for maintaining the delicate balance between excessive inflammation and defensive responses. Hepatic NKT cell accumulation is impacted by microbial bile acids, which mediate CXCL16 expression. Bile acids changed by Clostridium species raise the levels of CXCL16 in liver sinusoidal endothelial cells. Furthermore, hepatic NKT cell recruitment has demonstrated remarkable anticancer effects on EL4 lymphoma tumors (90, 91). The development of CD4+ T helper cells into subsets with a variety of effector roles that are best suited for host defense against invasive pathogens—such as Th1s, Th2s, Th17s, Tfhs, and iTregs—is a critical step in the adaptive immune response process (92). Immune system homeostasis is largely dependent on the balance among these subtypes.

#### 3.2.2 Regulation of gut microbiota Th1 cells

Th1s and Th2s mediate distinct immune responses on the basis of their unique expression patterns of cytokines and transcription factors (93). Th1 cell responses are induced in the gut by the Klebsiella (K.) genus, which includes *K. aeromobilis* and K. pneumoniae. The colonized Klebsiella treatment of GF mice intestines promotes Th1 cell proliferation and results in Th1 cell augmentation in the intestine (94). It has also been demonstrated that probiotic bacteria alter Th1 cell function. Th1 cells are closely connected to the probiotic Lactobacillus strains that are used. *Lactobacillus (L.) plantarum* (95) and *L. salivarius* (96) enhanced the production of Th1 cytokines, tumor necrosis factor alpha (TNFα), and interferon gamma (IFNγ). Furthermore, through the activation of macrophages, lactobacillus strains isolated from fermented foods increase TNFα release while concurrently decreasing levels of the Th2 cytokine interleukin (IL)-4 (97). Th2 cells release IL-4, IL-5, and IL-13, which are important components of humoral immunity, helminth infection defense, and the development of chronic inflammatory disorders such allergies and asthma (98). It has been found that some strains of Lactobacillus and *B. fragilis* suppress Th2 activity by enhancing Th1 activity first (97, 99). Th17 cells are responsible for the production of IL-17, a strong proinflammatory cytokine that damages tissue and plays a role in the etiology of autoimmune and inflammatory diseases (100).

#### 3.2.3 The gut microbiota and Th17 cells

Th17 cells are T cells that are highly proinflammatory and implicated in immune clearance from extracellular fungi and bacteria (101) and play a critical role in the intestinal mucosal immune defense system. Th17 cells also use class switch recombination to stimulate B-cell proliferation and antibody production (102), It is necessary for the development of tertiary lymphoid follicle-like structures and ectopic lymphoid follicles in target organs (103, 104). Th17 differentiation is generally induced by the synergy between transforming growth factor-β (TGF-β) and IL-6, and the development and maintenance of Th17 cells are supported by IL-23 (105). Through a number of methods, the gut microbiota promotes the constitutive production of Th17 cells in the LP (106). Initially, the combination of IL-6 and TGF-β to enhance Th17 development is only activated when DCs identify and phagocytose infected apoptotic cells (107). Another important indicator of Th17 induction is the adherence of commensal bacteria to ECs. SFB is one of the most potent Th17 inducers among symbionts (108, 109). According to Koji and Ivanov, gut microorganisms control the development of Th17 cells. Following investigations, it was shown that gram-positive segmented filamentous bacteria (SFB), also known as “*Candidatus Arthromitus*,” stimulate the development of Th17 cells and the release of various cytokines, including IL-17 and IL-22 (110, 111). Prevotella is an additional bacteria that causes strong Th17 cell and cytokine release in the colon of mice (112).

#### 3.2.4 Regulatory effect of the gut microbiota on Tregs

Treg cells are essential for immune response modulation, immunological homeostasis, and immune tolerance to self-and nonself-innocuous antigens, all of which contribute to the prevention of autoimmune disorders (113). Treg cells are similarly impacted by Bifidobacterium strains, and it has been demonstrated that Bifidobacterium (Bi.) infantis and *Bi. Bifidum* operate to stimulate the production of Treg cells (114). *Lacticasei bacillus casei* stimulates the growth of Treg cells and the release of IL-10, while Lactobacillus strains are involved in the differentiation and activity of Treg cells (115). By targeting the majority of immune cells, tregs can either directly or indirectly induce immunological tolerance through contact-dependent mechanisms, immunomodulatory cytokines (such as IL-10, TGF-b, and IL-35), or metabolic disruption of target cells (116). Tregs are divided into two major categories inside the host. Induced regulatory T (iTreg) cells are generated *de novo* mostly by naïve conventional T cells in the intestinal immune niche, while CD4+CD25+Foxp3+ natural regulatory T (nTreg) cells develop from immature precursor cells in the thymus (117). The majority of intestinal Tregs (iTregs) are separated into two categories based on the expression of extra TFs. The first subset consisted of the colon’s predominant RORg+ Treg cells. These cells are important regulators of Th17 and group 3 innate lymphoid cells, and they also express the nuclear hormone receptor RORg and the transcription factor c-Maf (118). The second subtype of Helios- and Gata3-expressing cells were Helios+ Treg cells, which are primarily seen in the small intestine and are associated with pathways relevant to IL-33 (119). According to research, colonic Treg induction can be effectively induced by any one of the five Bacteroides species (*Bacillus intestinalis, Bacillus caccae, Bacillus thetaiotaomicron, Bacillus vulgatus, and Bacillus massiliensis*) (120).

## 4 Short-chain fatty acid microbial metabolites

SCFAs are saturated fatty acids with chain lengths ranging from one to six carbon atoms and are the main products of the fermentation of dietary fiber in the colon (121). The gut produces between 500 and 600 mmol of SCFAs each day, depending on the diet’s fiber level (122). Acetate (C2), propionate (C3) and butyrate (C4) are the most abundant SCFAs in the human body and the most abundant anions in the colon (123). After intraluminal injection in an isolated rat colon loop, acetate and butyrate, but not propionate, enhanced mucin secretion. Butyrate altered the expression of the MUC2 gene, which in turn promoted the formation of mucin in the colon of mice (124). Some bacteria, including *Faecalibacterium prausnitzii, Roseburia intestinalis*, and *Anaerostipes butyraticus* (125), ferment to break down complex carbs and produce short-chain fatty acids (SCFAs) (6), which control the immunological system of the host and provide colonocytes with carbon (7). As a fundamental part of the innate immune system, intestinal epithelial cells (IECs) use both passive and active mechanisms to identify and ingest stem cell factor (SCFAs), which have an impact on the intestinal milieu. The majority of ingested butyrate is metabolized by IECs, whereas the liver mostly absorbs propionate, and larger amounts of acetate enter the bloodstream. The production of transforming growth factor β (TGF-β) in IECs is induced by butyrate derived from commensal bacteria. This process is mediated by the inhibitory effect of butyrate on histone deacetylase (HDAC) and through transcription factor-specific protein binding to the core promoter. This leads to the expression of TGF-β1 in IECs and the subsequent convergence of Tregs in the gut (126, 127). The gut microbiota, particularly butyrate-producing bacteria, ferments fibers into fermentation products such as SCFAs when the gut is in a homeostatic state. The activation of mitochondrial oxidation is dependent on peroxisome proliferator-activated receptor gamma (PPAR-γ), which is stimulated by these SCFAs. This results in a decrease in epithelial oxygenation. Additionally, SCFAs, including GPR109A, GPR41, and GPR43, which reduce inflammation in the gastrointestinal tract, directly bind G protein-coupled receptors (GPCRs) on the surface of immune cells and epithelial cells. When SCFAs enter host cells by diffusion or transport, their metabolism occurs, and/or histone deacetylase (HDAC) activity is inhibited (128). Enterobacteriaceae use virulence factors to induce neutrophil transepithelial migration during dysbiosis of the gut. This migration reduces the luminal concentration of short-chain fatty acids, including butyrate, by depleting SCFA-producing bacteria. The ensuing metabolic reprogramming of the epithelium increases lactate and oxygen (O2) bioavailability in the lumina (128).

## 5 Gut microbiota dysbiosis in human disease

Dysbiosis of the human gut microbiome has been linked to a variety of diseases (22). Studies on humans and animals have shown that gut dysbiosis—indicated by differences in the variety and frequency of the microbial population that makes up the gut flora—is associated with immunological dysregulation, aberrant brain protein aggregation, inflammation, and decreased neuronal and synaptic maturation (129). The intestinal microbiota has been shown to play a major role in both health and disease based on the findings of current epidemiological, physiological, and omics-based investigations as well as cellular and animal studies (130). Although the field of study on the complex gut microbiota is still in its early stages and there is still a lack of understanding about its functional characteristics, some encouraging studies have revealed that they point to an enormous opportunity for revolutionizing both therapeutic approaches and the pathophysiology of diseases (130–132). Several studies have provided evidence in favor of the concept that the gut microbiota regulates immunity, energy homeostasis, weight gain or loss, and illnesses associated with obesity (133).

Food supplements and diets have a large impact on the microbial makeup of the gut and how it changes over time. High-fat diets increase the risk of developing conditions, including diabetes, metabolic syndrome, and obesity, all of which are connected to notable alterations in the composition of the gut microbiota. The probability of intestinal dysbiosis increases when the circadian physiological rhythm is disturbed, and this may contribute to the etiology of a number of inflammatory and metabolic diseases, including cancer, diabetes, and intestinal inflammatory diseases (134). Dysbiosis of the gut, or unfavorable changes in the makeup of gut microbes, can lead to inflammation, oxidative stress, and insulin resistance in addition to weakening the mucosal barrier and dysregulating immunological responses. The translocation of bacteria and their metabolic products across the mucosal barrier, as well as chronic dysbiosis of the gut, can lead to a rise in the prevalence of several disorders. An altered gut microbiota has been linked to several serious human diseases, including obesity, diabetes, hypertension, and neurological and neurodevelopmental abnormalities (22, 130). Similarly, several nonalcoholic fatty liver diseases (NAFLDs), inflammatory bowel disorders (IBDs), hepatocellular carcinoma, cardiovascular diseases (CVDs), alcoholic liver disease (ALD), chronic kidney diseases (CKDs), and cirrhosis are associated with gut microbiota and its metabolites (135–137). A state called “dysbiosis” refers to the variation in the gut microbiota composition, which causes many diseases, including inflammatory bowel disease (IBD), diabetes, obesity, neurological disorders, cardiovascular disease, colorectal cancer, and autoimmune diseases as shown in **Table 1**.

**Table 1:** Gut microbiota dysbiosis and associated human disease.

Disease	Causation vs bacteria that decrease	Changes in microbiota or increase in number	Reference
Colorectal cancer	Prevotella, Ruminococcusspp., Pseudobutyrivibrio ruminis	Acidaminobacter, Phascolarctobacterium, Citrobacter farmer	(138)
Liver disease	Alistipes, Bilophila, Veillonella, Faecalibacterium, Ruminococcus, Bifidobacterium, Prevotella, Coprococcus, Veillonellaceae, Prevotellacopri, Faecalibacterium, Haemophilus	Claustridum, Bacteroidetes, Betaproteobacteria, Lactobacillus., Collinsella, Corynebacterium, Prevotellaceae, Ruminococcus, Sarcina, Sutterellaceae, Enterobacteriaceae, Bacteroidaceae	(139)
Diabetes: Diabetes type1	Lactobacillus, Bifidobacterium, Blautia coccoides, Eubacterium rectal, Prevotella, Firmicute	Clostridium, Bacteroides, Veillonella	(140)
Diabetestype2	Firmicutes, Clostridia, Lactobacillus, Eubacteriumrectale	Bacteroids-PrevotellaVerses Clostridiacocoides, Betaproteobacteria, Bacteroidetes/Firmicutes	(141)
HIV	Clostridia, Bacteroidia, Lactobacilli, Bifidobacteria	Erysipelotrichaceae, Proteobacteria, Enterobacteriaceae, Candidaalbicans Pseudomonas aeruginosa	(142)
Alzheimer disease	Probiotic treatment did not meaningfully change other factors including oxidative stress and inflammation, but it may have a good impact on AD patients’ cognitive function.	probiotic supplementation containing: *Bifidobacterium bifidum Lactobacillus casei*, *Lactobacillus fermentum and Lactobacillus acidophilus*,	(143)
Parkinson disease	When mucosal and stool samples with Parkinson’s disease, several genes were shown to be downregulated in the stool microbiota of these people; the microbiota composition of the mucosal and stool samples was linked to substantial changes in patients with PD.	Bacterial increase: Proteobacteria, Betaproteobacteria, Coprococcus, Blautia, Akkermansia, Oscillospira, Roseburia, Bacteroides; bacterial decrease: Faecalibacterium, Firmicutes, class Clostridia	(144)
Parkinson disease	Change in the fecal microbiota may contribute to the development of PD; Prevotellaceae was decreased in people with Parkinson’s disease, and a high abundance of this genus was not indicative of having PD; Prevotellaceae may serve as a biomarker to rule out PD because of their great abundance.	Bacteria decrease, Provotellaceae; the abundance of Ruminococcaceae could be associated with levels of Provotellaceae	(145)
Autism	Children with autism have higher concentrations of Suterella spp. in their feces, and Ruminococcus torques is also more prevalent and may be linked to GI issues in these kids.	Bacteria increase: *Ruminococcus torques* and *Suterella*	(146)
Autism	A less diversified microbiome was found in autistic children, and the intestinal microbiota was linked to GI problems. Bacteria reduction: Veillonellaceae Coprococcus and Prevotella; main phyla in the microbiota of patients with autism: Bacteroidetes and Firmicutes;	most rich genera: Akkermansia, Bifidobacterium, Bacteroides, Faecalibacterium, and Subdoligranulum	(147)
Autism	Detected a connection between bacterial populations and genes expressed in the colon of autistic children; the source of these intestinal abnormalities is still under investigation. Bacteria decrease: Bacteroidetes	Bacteria increase; Bacteroidetes, Firmicutes, Lachnospiraceae and Ruminococcaceae, Betaproteobacteria,	(148)
Depression	GF animals colonized with a “depression microbiota “had additional symptoms compared to control GF animals. Bacteria reduction: Acidaminococcaceae, Rikenellaceae, Lachnospiraceae, Veillonellaceae, Bacteroidaceae, and Sutterellaceae	Lactobacillaceae Coriobacterineae, Clostridiales, Streptococcaceae, Actinomycineae Lachnospiraceae Erysipelotrichaceae, Ruminococcaceae, and Eubacteriaceae:	(149)
Depression	An improved understanding of the association between the microbiota and occurrence of particular bacteria with symptoms associated with depression resulted from examination of fecal samples from people with and without depression. Bacteria lessening: Prevotellaceae Erysipelotrichaceae, Lachnospiraceae., Veillonellaceae Bacteroidaceae, and Ruminococcaceae	Rikenellaceae Enterobacteriaceae, Acidaminococcaceae, Porphyromonadaceae, and Fusobacteriaceae,	(150)
Anxiety	Probiotic administration has been linked to better mental health; nevertheless, this probiotic combination had no negative effects on the hypothalamic−pituitary−adrenal (HPA) axis.	Probiotic supplement *Lactobacillus casei*, *Bifidobacterium logum*, *LA5* and *Bifidobacterium lactisBB12, Bifidobacterium breve*, *Lactobacillus acidophilus, Lactobacillus rhamnosus*, *Lactobacillus, thermophilus*, *bulgaricus Streptococcus*	(151)

A substantial amount of immune cell activation and cytokine release during severe trauma cause microvascular constriction, tissue hypoxia, and a significant amount of neutrophil activation and aggregation, which are subsequently coupled with adhesion molecules. It increases the permeability of capillaries and allows a high number of inflammatory cells to infiltrate distant organs by acting on the surface of endothelial cells (9). Proinflammatory and anti-inflammatory chemicals are in dynamic equilibrium during the physiological response stage, and the immune system’s inflammatory response is largely stable to prevent an overly strong inflammatory response or immunosuppression and to lessen additional trauma-related tissue damage (152). The gut microbiota controls the development and operation of immune cells that live in the central nervous system, including microglia (153), and influences peripheral immune cell activity, which controls the immune response of the central nervous system (154). The complicated ecosystem known as the gut microbiota is necessary for tissue healing, nutrient metabolism, and host defense against infection (155). Food-related sensory stimulation is absent from enteral nutrition delivery, although it can modulate the intestinal flora to control the inflammatory response. After trauma, the body needs more energy than usual, which is reflected in a high metabolic state. Several trials have demonstrated that emulating feeding increases salivation, efficiently stimulates gastrointestinal motility, removes oral germs and viruses, and significantly improves depressive symptoms (156, 157).

## 6 Trauma

Trauma is the term used to describe the destruction of tissue structure, dysfunction, and/or psychological damage caused by environmental, biological, chemical, physical, or psychological factors (158). Physical trauma consists of injury to the body, such as from accident, fall or violence, while the psychological trauma consists of emotional and mental injury resulting from extreme stress or shock, such as abuse, neglect or witnessing a traumatic event.

### 6.1 Traumatic brain injury and gut microbiota

Numerous preclinical and human investigations have been done to understand how traumatic brain injury (TBI) affects dysbiosis and the intestinal microbiota. Acute fecal microbiome studies following traumatic brain injury show alterations in bacteria composition and diversity compared to controls 24 hours after injury (159). Hou et al. demonstrated changes in microbial composition, variations in beta diversity at days 3 and 7, and a decrease in alpha diversity at 3 days after injury in male rats with TBI (160). Bao et al. reported modifications in the microbial composition but failed to find any abnormalities in alpha- or beta-diversity seven days after damage in a male mouse model of TBI (161). Nicholson et al. examined the microbiome at two hours24 hours, 3 days, and 7 days following traumatic brain injury, they discovered acute reductions in alpha-diversity by day three, shifts in beta-diversity at days one and three, and modifications in the makeup of microorganisms (162). In the same way, a male mouse model of TBI demonstrated a significant drop in the Chao1 index one hour after damage, which continued for a week along with changes in beta-diversity and microbial composition (163). In a different study, male rats with traumatic brain injury (TBI) showed sustained abnormalities in beta-diversity at 4, 30, and 60 days after injury, although microbiome alterations in terms of alpha-diversity at 3 days vanished after 1 month (164). Zhang et al. showed jejunal injury only 2 hours after TBI and hemorrhagic shock in rats, with evidence of caspase-1-positive cells and pyroptotic cells which resolved by day 30 (165). When male mice were given a TBI, examination of the small intestine revealed villous reduction, a decrease in Paneth cell lysozyme present, and an increase in caspase-3 levels, which are suggestive of apoptosis (159). Some investigations looked into possible links between dysbiosis and bone formation, post-traumatic epilepsy, neurologic sequelae, and post-traumatic stress disorder in light of the results of microbiome changes following traumatic brain injury. According to the study, post-traumatic epilepsy can be categorized according to the makeup of microbes (166). One day after TBI, a mouse study revealed an abrupt drop in the expression of neuronal markers in the duodenum and colonic tissue, which may have an impact on intestinal peristalsis (167). A study reported 34 patients with moderate or severe traumatic brain injury (TBI) were compared to 79 patients without TBI, 297 people with no TBI, and one of the biggest human investigations of the fecal microbiota post TBI (168). Brenner et al. collected samples from patients who suffered from moderate to severe traumatic brain injury (TBI) an average of 28 years after the injury. They found no differences in the microbial composition, alpha-diversity, or beta-diversity between these patients and those who had no or mild TBI, indicating that dysbiosis following TBI resolves at this later stage (168).

### 6.2 Spinal cord injury and gut microbiota

Spinal cord injury (SCI) has also been studied to evaluate gut dysbiosis postinjury, although mainly in female animal subjects. Kigerl et al. performed T9 spinal cord contusion in female mice, they saw a drop in Bacteroidales and an increase in Clostridiales in the fecal microbiome at five distinct intervals over the process of 28 days following injury (169). A contusion at the T8–10 level caused spinal cord injury in female mice, which, two weeks after injury, showed reduced abundance of Bacteroidetes and increased Firmicutes and Proteobacteria and differences in beta-diversity and microbial composition. The sham groups only underwent laminectomy (170). The study demonstrated increased expression of proinflammatory factors interleukin-1 beta and interleukin-12 in the small intestines of injured animals after four weeks (171). A study of T10 spinal cord contusion in mice showed beta-diversity differences between injured mice and controls along with different makeup of microbes at seven days postinjury (172). Studies on humans have also been conducted to assess how spinal cord damage affects the microbiome, revealing changes in its diversity and composition. Within 60 days of the injury, Baz Zocchi et al. examined the intestinal microbiota of 100 patients and found no variations in alpha-diversity, although they did find changes in beta diversity (173). Along with characterizing the microbial makeup of these patients, they also noted that the degree of dysbiosis varied according to the degree and completeness of spinal cord injury, and that patients with spinal cord injury had high concentrations of Methanobrevibacter, Streptococcus, Enterococus, Klebsiella, and Akkermansia (173). Compared to healthy controls, Zhang et al. observed a decrease in alpha-diversity (Simpson), changes in beta-diversity, and an abundance of genera like Bacteroides, Blautia, Lachnoclostridium, and Escherichia-Shigella in the fecal microbiome of 43 male patients with complete spinal cord injuries at least six months after the injury (174). Patients who suffered acute SCI had an abundance of Sutterella and Odoribacter whereas long-term SCI patients had more Clostridiales (175). At an average of 5.6 months after the injury, Yu et al. examined the intestinal microbiome of 21 patients with complete SCI and 24 patients with incomplete SCI. They discovered that both groups’ shifts in beta-diversity were similar to those of healthy controls, but that complete SCI patients’ shift was more pronounced than that of incomplete SCI patients (176). Lactobacillaceae, Lachnospiraceae, Eubacterium, Clostridium, and Sutterella were abundant in incomplete SCI patients, while Coriobacteriaceae, Syngergistetes, Eubacterium, and Cloacibacillus dominated the gut microbiomes of complete SCI patients (176). Over all studies demonstrated that the level of SCI was correlated with gut microbiota profiles.

### 6.3 Multiple trauma and gut microbiota

Several studies on animals have been carried out to comprehend the potential effects of multiple traumas on the intestinal barrier and microbiome. In animal research, more severe models of multiple injuries, known as polytrauma, have shown both acute and long-term dysbiosis. Nicholson and colleagues evaluated male rats that underwent a polytrauma model consisting of a femur fracture, hemorrhagic shock, and crush injuries to the liver, small intestine, and skeletal muscle of an extremity. The fecal microbiota was assessed two hours after the injuries (177). This group observed that whereas alpha-diversity remained unchanged, beta-diversity drastically changed along with changes in the microbial makeup; injured rats had high Lachnospiraceae abundances (177). A different study of multiple injuries in male rats—a laparotomy, crush injuries to the liver and skeletal muscles, and a femur fracture with hemorrhagic shock—showed differences in beta-diversity and changes in the composition of the microbiome, with higher levels of Roeburia and Enterobactericeae and lower levels of Rothia and Streptococcus. However, no significant differences in alpha-diversity were found at this early stage (178).

Several studies have been conducted in humans to understand the effects of multiple and severe injuries on the intestinal microbiome. Burmeister et al. studied 67 trauma patients with an average injury severity score (ISS) of 21 and performed a rectal swab on admission and characterized the microbiome acutely postinjury (179). This group identified that higher alpha-diversity was correlated with increased survival; in addition, shifts in beta-diversity postinjury were correlated with body mass index, sex, length of hospital stay, length of intensive care unit stay, and mortality. This study also correlated decreased Firmicutes and blooms of Prevotella and Corynebacterium in the intestinal microbiome on admission with survival (179). Nicholson et al. studied 72 patients admitted after severe injury with an average ISS of 21 and evaluated changes in the microbiome with serial samples taken over the course of almost two weeks (180). These results revealed a rise in alpha-diversity at the beginning of the hospital stay, but by day five, there was a noticeable decline that lasted for nearly two weeks without any recovery (180). Similar to this, these individuals experienced sustained beta-diversity alterations upon admission, which were even found to be connected with the severity score of their injuries (180). In addition, compared to healthy controls at all times, trauma patients in this study displayed a distinct microbiome profile marked by an abundance of Proteobacteria and a depletion of Firmicutes (180).

Studies investigated into several treatments to lessen this dysbiosis in light of the compelling evidence showing the formation of a pathobiome following a single or a series of injuries. Pre-injury inulin administration was studied for two months in the same murine model, and the results showed a change in beta diversity in the cecal contents at 24 hours, 1.5 months, and three months after the injury, along with a decrease in pathogenic bacteria and an increase in bacteria that produce short-chain fatty acids (181). In mice with traumatic brain injury, Li et al. provided Clostridium butyricum, a probiotic that produces butyrate, for two weeks prior to and two weeks following the injury. The results showed that the treatment preserved colonic occludin expression and reduced the expression of inflammatory markers in the colon. Additionally, the treated mice showed fewer brain edema, less neurologic degeneration, and less neuronal apoptosis (182). Ma et al. revealed enhanced alpha-diversity and restoration of microbial composition coupled with neuroprotective effects after giving Lactobacillus acidophilus to mice for one, three, or seven days following traumatic brain injury (183). In a study by Jing et al., they used melatonin to treat a spinal cord contusion in a murine model. After four weeks, the treated mice showed improved postinjury locomotor testing, restored commensal bacteria in the intestinal microbiome, and had decreased intestinal permeability with increased expression of zonulin and occludin in the colon (184). Hou et al. studied a model of brain injury in male rats and showed that administration of brain proteins and Lactobacillus and Bifidobacterium probiotics resulted in elevated alpha-diversity and decreased intestinal permeability compared to untreated counterparts in addition to decreased circulating inflammatory cytokines within two weeks of injury (160). In a study conducted by Brenner et al., 16 TBI patients were given Limosilactobacillus reuteri daily for 8 weeks. The patients’ plasma C-reactive protein levels were found to decrease with this probiotic, but there were no differences in alpha- or beta-diversity or microbial composition when compared to the placebo group (185).

### 6.4 Immune response to traumatic injury

Traumatic injury initiates concurrent systemic inflammatory response syndrome and anti-inflammatory compensatory anti-inflammatory response syndrome (CARS) (186), caused by tissue damage and hemorrhagic shock, which triggers the release of damage-associated molecular patterns (DAMPs) from necrotic and injured cells. Through the activation of pattern recognition receptors (PRRs), DAMPs activate immunological and complement cells (9), causing the cytokines that initiate the systemic inflammatory response to be released to progress (187). Additionally, there is growing evidence that links DAMP levels to immunological suppression after surgery and acute injury (188). High-mobility group box protein 1 (HMGB1) is a type of DAMP that has been demonstrated to be elevated in the blood after trauma (189), in nuclear and mitochondrial DNA, and in heat shock protein 13 (190). The function of DAMPs in trauma has been thoroughly studied elsewhere (191). Within one hour of injury, trauma patients’ plasma has noticeably higher levels of HMGB1, the most extensively researched DAMP after trauma (191), with levels according to the severity of the injury, the start of sepsis, and Multifil organ dysfunctions syndrome (MODS) (189). In immune cells, HMGB1 has a variety of proinflammatory effects, including enhancing cytokine release, reactive oxygen species (ROS) generation (192), and chemotaxis (193). Additionally, HMGB1 directly impacts the vascular endothelium by promoting neutrophil adherence, elevating endothelial permeability, and upregulating the production of adhesion molecules and cytokine release (194, 195). DAMP-induced cytokine production is thought to be a major initial component of the systemic inflammatory response to trauma (196). Increased levels of proinflammatory cytokines/chemokines, such as interleukin (IL)-1b, IL-6, IL-8, granulocyte colony stimulating factor (G-CSF), and tumor necrosis factor a (TNFa), have been reported in a number of investigations involving critically injured individuals (188, 197). These alterations appear quite quickly following the original injury and continue in the hours and days that follow (197, 198). The levels of numerous anti-inflammatory mediators, including transforming growth factor-b1, the IL-1 receptor antagonist, and IL-10, also increase in the circulation at the same time (198). Compared to patients without multiple organ dysfunction syndrome (MODS), patients with MODS have higher pro- and anti-inflammatory cytokine levels upon hospital admission (197, 198). Interestingly, whole blood from trauma patients treated with lipopolysaccharide (LPS) produced fewer cytokines/chemokines than whole blood from healthy controls. This could lead to increased susceptibility to infection following severe damage (199). Major modifications in neutrophil function and phenotype are also carried out by traumatic injury (200). Patients with severe injuries have been found to have surface expression of CD62L (lower) and CD11b (higher), which are indications of neutrophil activation (199). Decreased effector functions (such as phagocytosis and responsiveness) and immunosuppressive neutrophils may be responsible for trauma patients’ increased susceptibility to infection (201). Similarly, there is a rise in lymphocyte counts during the hyperacute (<2 h) window after injury (202), and activation of the former is primarily linked to notable increases in the populations of CD4+, CD8+, and circulating natural killer (NK) T cells (202). However, over time, the level of lymphocytes in circulation decreases, and as early as four hours after injury, significant lymphopenia has been observed in a number of studies involving trauma patients (202, 203). Reductions in CD4+, CD8+, NK, and γdT-cell populations have been observed, and these alterations in T-cell numbers rather than B-cell numbers are responsible for the overall decrease in lymphocyte number (199, 202). Despite tremendous progress, there are still many unknowns regarding the immunological response to severe injury. Preclinical animal models are essential for improving our fundamental understanding and creating new treatment approaches that target posttraumatic conditions such as sepsis and multiple organ dysfunction syndrome (MODS), as clinical research can be complex, suggesting that early intervention might be important (204). Blood products are considered to provide numerous advantages for severely injured patients, including improving tissue perfusion, replacing lost clotting factors, and restoring endothelial function. They are regarded as a crucial component of damage-controlled resuscitation (205). However, the impact of these resuscitation products on the immune system’s reaction to trauma is still mostly unclear.

## 7 Therapeutic implications of gut microbiota

The gut microbiota mediates the efficacy and toxicity of chemotherapy and immunotherapy. The gut microbiota is to be used as biomarkers to predict treatment response or adverse reactions and, at the same time, to be modulated for improving treatment and patient outcomes (206). Probiotics are live bacteria that affect the gut microbiota of the host to produce positive effects (207). Two of the most commonly used probiotic bacteria, Lactobacillus and Bifidobacterium species, have been shown to enhance mucosal trophic effects by inducing responses from the intestinal epithelial cell barrier, inhibiting pathogen colonization, stabilizing the preexisting microflora, and improving immune system responses (208, 209). The Bifidobacterium breve probiotic strain (CCFM1025) has demonstrated positive effects on reducing gastrointestinal and psychological abnormalities in patients with major depressive disorder (MDD) (210). Lactobacilli and Bifidobacteria, two well-known probiotic strains, play key roles in maintaining gut and mental homeostasis (211). Because of their psychotropic characteristics, lactobacilli and bifidobacteria are classified as psychobiotics because they can enhance behavior in people who are depressed or anxious (211, 212). The Lactobacillus plantarum strain PS128 has been shown to reduce anxiety-like behavior and sadness in tested animals according to a study by Liu et al. on adult mice with and without early stress induction (212). According to research by Tao et al., the probiotic Lactobacillus GG releases soluble molecules that stimulate the p38 MAPK pathway to synthesize heat-shock proteins, thereby protecting intestinal epithelial cells from damage (213). Another study revealed that in patients with mild to moderately active ulcerative colitis (UC), VSL#3, a high-potency probiotic medical food comprising eight different strains, could cause remission and prevent inflammatory disease relapse (214, 215). Other probiotics, such as *Bifidobacterium bifidum*, *L. acidophilus* (216), and L. reuteri ATCC55730 (217), have also been associated with positive outcomes in IBD patients according to previous reports. Furthermore, the benefits of probiotics have been linked to the restoration of goblet cell quantity and function as well as the induction of protective immunoglobulin secretion by the mucosal immune system in the intestinal tract, including secretory IgA, protective defensins, and bactericidins (218). Pre- and probiotic formulations used as food supplements that are also used as psychobiotics must adhere to the quality requirements of the World Health Organization legislation. These requirements include the need for formulations to contain specific microbial strains that are sufficiently characterized, safe for intended use, supported by positive results from human clinical trials, designed in accordance with scientific standards or recommendations from local/national authorities, and, last but not least, viable and effective at the appropriate dose during storage (219, 220). Several types of pre- and probiotic preparations or novel foods might be considered possible formulations considering their influence on disease incidence.

Emerging evidence suggests that gut microbiota can mediate the anticancer effects of some chemotherapeutic agents, including 5-fluorouracil (221), cyclophosphamide (222), gemcitabine (223), through several mechanisms such as microbial translocation, immunomodulation, metabolism, enzymatic degradation, and reduced ecological diversity (224). Since experimental research indicates that *F. nucleatumcan* triggers autophagy to confer resistance to oxaliplatin and 5-fluorouracil, the role of gut microbiota in chemotherapy resistance has also been examined (225). The gut microbiota regulates the metabolism and side effects of irinotecan (CPT-11), a topoisomerase inhibitor prodrug of SN-38 that is frequently used to treat colorectal cancer (226). The gut microbiota is required for the effective immune response in immunotherapy (227), and can affect the response to immune checkpoint inhibitors targeting the programmed cell death 1 (PD-1)–programmed cell death 1 ligand 1 (PD-L1) axis (227, 228) and the cytotoxic T lymphocyte-associated antigen 4 (CTLA-4) axis 228. Specific bacteria were positively correlated with immunotherapeutic response, including *Akkermansia muciniphila* (227), *Bifidobacterium* spp. (228), *Eubacterium limosum* (229), and *Alistipes shahii* (230). Importantly, oral gavage of *A. shahii* reconstituted the immunotherapeutic response against colon tumors in antibiotic-treated mice (230). In a study that combined shotgun metagenome data from three studies on anti-PD-1 antibody response, enrichment of *A. muciniphila* and *Ruminococcus champanellensisin* responders to immunotherapy was observed despite differences in the primary studies (231). Probiotics restore the gut microbiota dysbiosis caused by acute CNS injury, which greatly improves brain injury. One possible target to help treat acute CNS damage is the gut microbiome (232). Traumatic injury is linked to a GM that has a higher concentration of several different commercially available probiotic species, such as *Eubacterium biforme*, *Oxalobacter formigenes*, and *Akkermansia muciniphilia*, but less unique organisms overall. When it comes to therapeutic and diagnostic targets for traumatic injury, the GM is quite promising (179). This finding has led to the possibility of detecting these core bacteria as predictive biomarkers for immunotherapy response.

## 8 Challenges and limitations in immune-microbiome research

Recent research has greatly enhanced our understanding of the intimate but complicated crosstalk between the microbiome and the immune system. Nevertheless, many unknowns and challenges remain, in disentangling microbiome immunity interactions in homeostasis and disease (43). Different microbiota and metabolites are responsible for immune activation, while on the other hand, persistent inflammation can influence the dysbiotic structure and roles of microbial communities. In most medical disorders, however, a direct causal association between the microbiome and immunity prior to or during the early stages of disease has not been established (43). Further research is needed to fully understand the significance of other yet underestimated microorganisms, such as viruses, fungi, and parasites, and how they affect host immunity. Furthermore, a combination of genetic and environmental factors (such as nutrition, smoking, etc.) impact the development of numerous diseases with uncertain etiologies, such as cancer, autoimmune arthritis, and IBD (233). The relationship between the immune system and the microbiota in the context of host genetics and environmental stimuli must be thoroughly studied. It will be possible to better understand how the gut microbiota and the immune system are cross-regulated in these various and complicated circumstances by integrating multiomics data sets, such as metagenomics, single-cell transcriptomics, epigenomics, proteomics, and metabolomics. Importantly, the microbiome research community mostly relies on laboratory mice in all of these endeavors, which has limited translational potential and reproducibility when compared to “real-life” situations because they have a different microbiota from “wild” animals and people (234).

The microbiota is currently only studied from one or a few aspects using methods like 16s rRNA amplicon sequencing for microbiota profiling or shotgun metagenomic WGS for WGS. In contrast, the human microbiota plays a complex, multifaceted role in the pathogenesis of disease. This role is characterized by interactions with the host that are both structural (such as the profiling of microbial composition) and functional (such as the identification of entire genetic pathways). Additionally, these interactions are mediated by a variety of small molecules, such as metabolites, catabolites, and signal molecules (235). The challenges specific to multi-omics include the variability of results resulting from different software pipelines and an over-reliance on the few publicly available and carefully curated microbiome databases, which restrict the information to well-characterized microorganisms, transcripts, proteins, and metabolites (235). Due to the several difficulties that contemporary microbiota research must overcome, our understanding of the intricate web of interactions between resident bacteria and the host is still restricted. Budgetary constraints have prevented most research from fully analyzing the human microbiota from a compositional perspective. This has resulted in a dearth of knowledge regarding the complex network of genes, proteins, and metabolic processes that may be necessary to determine the microbiota’s etiopathogenetic role (236). There are still significant obstacles to be addressed in this area, including the need for more sophisticated and all-inclusive bioinformatic tools to be made more widely available so that non-bioinformaticians can also benefit from the information that can be gleaned from meta-omics data and for the cost of analysis per sample to be lowered in order to make the data more accessible. Additionally, the lack of standardization in sample collection from various locations, transportation, and storage, along with the use of heterogeneous methods for genomic DNA extraction, may introduce bias into the results by adding to the variability in the quality of bacterial DNA isolation and partially removing contaminating host DNA.

## 9 Conclusions and future directions

Both clinical and preclinical studies have suggested that a large number of immunological mechanisms are linked to the gut microbiota and a large number of human diseases. Preclinical and clinical trials should be conducted to better understand how microbiota can interact with human immune system and disease. Dysbiosis can be caused on by an unhealthful diet, which includes taking too many antibiotics and consuming little fruits and vegetables. The functional importance of intestinal dysbiosis in maintaining intestinal homeostasis, inducing the intestinal epithelium’s immune response, and interacting with other factors via genetic or epigenetic mechanisms is making it an increasingly important risk factor. The intestinal microbiota is subjected to changes in both host and exogenous factors, but how dysbiosis is triggered and leads to chronic inflammation is largely unknown. Treatment of dysbiosis through fecal microbiota transplantation (FMT) or prebiotic administration has produced favorable results in clinical trials; however, both the safety and efficacy of these treatments must be determined before they can be considered therapeutic strategies. Targeting the gut microbiota and its metabolism for autoimmune diseases may be a promising therapeutic and diagnostic approach. Our understanding of immune responses to traumatic injury has rapidly increased in the last decade, but preclinical animal models have played a significant role in this progress. Future studies must clarify the underlying links between the GM and immune cell mechanism and response to trauma. Trauma itself has a greater impact on gut flora and immune cells. However, the mechanism is still unclear. Probiotics, including psychobiotics, are crucial in maintaining body homeostasis in all disorders that have been studied. In the future, additional research should focus on the impact of psychobiotics on the health status of patient age range, health issues, and genetic background and on single- and multi probiotic formulations, dosage, and time of administration. Future innovations may involve the creation of synthetic prebiotics and targeted probiotics that are specifically designed for an individual’s gut microbiota. Strategies to treat dysbiosis have centered on microbiota-based therapies, such as probiotics, prebiotics, precision editing of the microbiota, or fecal microbiota transplantation, since it was discovered that alterations in the microbiota composition can contribute to chronic human diseases. To find these metabolite-producing gut bacteria for medicinal uses, more research is needed. To provide more effective medications and diagnostics, more research should be done on the effects of prebiotic and probiotic use on human health. The probiotics, prebiotics, and postbiotics’ ability to alter the composition of gut microbiota and the immune system hold great promise as a new medical frontier. The gut microbiota to be used as biomarkers to predict the treatment of patient. These include methodological difficulties in determining the optimal biomarker combinations and thresholds, scientific difficulties in verifying biomarkers across various populations, and technical difficulties in creating a patient screening test that is both convenient and reasonably priced. Overcoming these challenges will require evaluating multiple biomarkers in different ethnic groups of patients to derive the best diagnostic algorithm across populations.

## Author contributions

HU: Conceptualization, Writing – original draft. SA: Validation, Writing – review & editing. YC: Writing – review & editing. QL: Writing – review & editing. CL: Writing – review & editing. YT: Writing – review & editing. KL: Funding acquisition, Supervision, Writing – review & editing.
